# Efficacy of deep sclerectomy using the transverse peeling technique: short-term outcomes

**DOI:** 10.1097/MS9.0000000000005338

**Published:** 2026-07-14

**Authors:** Ehab Y Alsirhy, Yahya A Alyahya, Musab K Alaql, Ahmed Z Alkhars

**Affiliations:** Glaucoma Research Chair, Ophthalmology Department, College of Medicine, King Saud University, Riyadh, Saudi Arabia

**Keywords:** deep sclerectomy, goniopuncture, intraocular pressure, STPT, trabeculo-Descemet membrane

## Abstract

**Purpose:**

Determination of the efficacy of Sirhy’s transverse peeling technique, a modification of traditional deep sclerectomy that involves the creation of a wider and longer deep scleral flap to increase aqueous humor drainage.

**Methods:**

This was a retrospective cohort study conducted on patients who underwent deep sclerectomy using the transverse peeling technique for the management of glaucoma. Complete success was defined as an intraocular pressure of ≤ 21 mmHg when the patient was off all intraocular pressure-lowering medications. However, qualified success was defined as an intraocular pressure of ≤21 mmHg when the patient was on additional intraocular pressure-lowering medication or laser goniopuncture. Failure was classified as an intraocular pressure of >21 mmHg at 6 months or the need for additional glaucoma interventions.

**Results:**

Forty-one eyes and 39 patients were included in this study. The mean preoperative intraocular pressure was 21.78 ± 7.62 mmHg, which was significantly reduced to 13.76 ± 3.95 mmHg postoperatively, with a complete success rate of 80.5%, a qualified success rate of 17.1%, and a failure rate of 2.4%.

**Conclusion:**

Deep sclerectomy using the transverse peeling technique is a novel approach that results in better control of intraocular pressure, a reduced need for post-operative antiglaucomatous medications, and a decreased requirement for goniopuncture.

## Introduction

Glaucoma is the leading cause of irreversible blindness worldwide and is characterized by progressive optic nerve damage, which is often associated with elevated intraocular pressure (IOP)^[^1,2^]^. In recent years, non-invasive surgical techniques have been developed to increase the safety of glaucoma surgery. Among these, deep sclerectomy (DS) has gained recognition as a well-established procedure, and an increasing amount of evidence supports its safety and effectiveness^[^3,4^]^. In DS, the aqueous outflow is increased by excising the inner wall of Schlemm’s canal and the juxtacanalicular trabecular meshwork, which are the primary sources of outflow resistance in open-angle glaucoma. In this procedure, the trabeculo-Descemet membrane (TDM) is preserved to regulate the aqueous flow through the filtration site. This controlled reduction in IOP contributes to the improved safety profile of DS and minimizes the complications associated with excessive drainage and hypotony^[^5–7^]^. The purpose of this study was to explore the efficacy of a novel technique called the Sirhy’s transverse peeling technique (STPT), which is a modification of traditional deep sclerectomy and involves the creation of a wide and smooth scleral flap to facilitate aqueous humor drainage.

HIGHLIGHTS

- This study highlights the efficacy of Sirhy’s transverse peeling technique (STPT), a novel modification of the traditional non-invasive deep sclerectomy.
- Deep sclerectomy using STPT results in better control of intraocular pressure and a reduced need for post-operative antiglaucomatous medications or surgical intervention.
- STPT achieved a complete success rate of 80.5% compared to 53.1–71.8% in previous studies.

## Methodology

A retrospective cohort study was conducted with patients who underwent deep sclerectomy using the transverse peeling technique for the management of glaucoma. This study was conducted in accordance with the ethical standards outlined by the Helsinki Declaration and was approved by the institutional review board (IRB) of the affiliated university. Informed consent was obtained from all patients who were enrolled in the ophthalmology outpatient clinic between 1 January 2020 and 1 February 2024. Patient data, including their demographic information, preoperative characteristics, surgical details, postoperative outcomes, and follow-up information, were collected from the electronic medical records of the hospital. Patients with a minimum of 6 months of follow-up were included, and the primary outcome was a reduction in IOP from baseline to postoperative follow-up visits. The secondary outcome measures focused on changes in the number of eye drops, the number of follow-up clinic visits, postoperative complications, and surgical interventions performed postoperatively. The postoperative follow-up schedule was day 1 and months 1, 3, and 6. Success was classified as complete, qualified, or failure at 6 months postoperatively. Complete success was defined as an IOP ≤ 21 mmHg when off all IOP-lowering medications, whereas qualified success was defined as an IOP ≤ 21 mmHg when on additional IOP-lowering medication or laser goniopuncture. Failure was classified as an IOP > 21 mmHg at 6 months or the need for additional glaucoma interventions. Deep sclerectomy using the transverse peeling technique was performed by the same surgeon (E.S.). All participants were prescribed topical ofloxacin 0.3% four times a day for 1 week and 1% topical prednisolone acetate (initially administered four times a day and gradually tapered over a 4-week period). The literature search was initiated on 1 January 2000 and included publications available up to 1 January 2025. Data analysis was performed using the Statistical Package for the Social Sciences, SPSS 23rd version. Frequencies and percentages were used to express the categorical variables. The minimum, maximum, mean, and standard deviation were used to express the numerical variables. A paired sample *t*-test was used to determine the significant differences in IOP before and after the intervention. The chi-squared test was used to determine associations between categorical variables. The threshold of significance was set at 0.05. Patients or the public were not involved in the design, conduct, reporting, or dissemination plans of our research.

## Results

A total of 39 patients and 41 eyes were included in this study. The minimum age in the study was 1 year, the maximum age was 85 years, and the mean age was 47.76 ± 26 years. Fifteen patients (38.46%) were males, and 24 (61.54%) were females. For laterality, 21 (51.22%) right eyes were included, and 20 (48.78%) left eyes were included. Among the patients, 30 (73.2%) had not undergone any glaucoma surgery previously, while 1 (2.4%) had undergone cyclophotocoagulation in the eye included in the study, 8 (19.5%) had undergone deep sclerectomy in the eye included in the study, and 2 (4.9%) had undergone endoscopic cyclophotocoagulation. A total of 21 (51.2%) patients had cataracts in the eye included in the study, and 19 (46.3%) patients had a history of cataract surgery in the eye included in the study. Regarding the type of glaucoma, 21 patients (51.2%) had primary open-angle glaucoma, 8 patients (19.5%) had secondary open-angle glaucoma, 8 (19.5%) had primary congenital glaucoma, 2 (4.9%) had juvenile open-angle glaucoma, and 2 patients (4.9%) had normal-tension glaucoma. Regarding preoperative medical management of the patients, 4 (9.8%) were on two topical medications, 35 (85.4%) were on full topical (three) medications, and 2 (4.9%) were on full topical medications and oral medications. In regard to the cup-to-disc ratio, one patient (2.4%) had a cup-to-disc ratio of 0.4 or less, 8 (19.5%) had a cup-to-disc ratio of 0.5–0.7, and 32 (78%) had a cup-to-disc ratio greater than 0.7.

**Figure 1.**
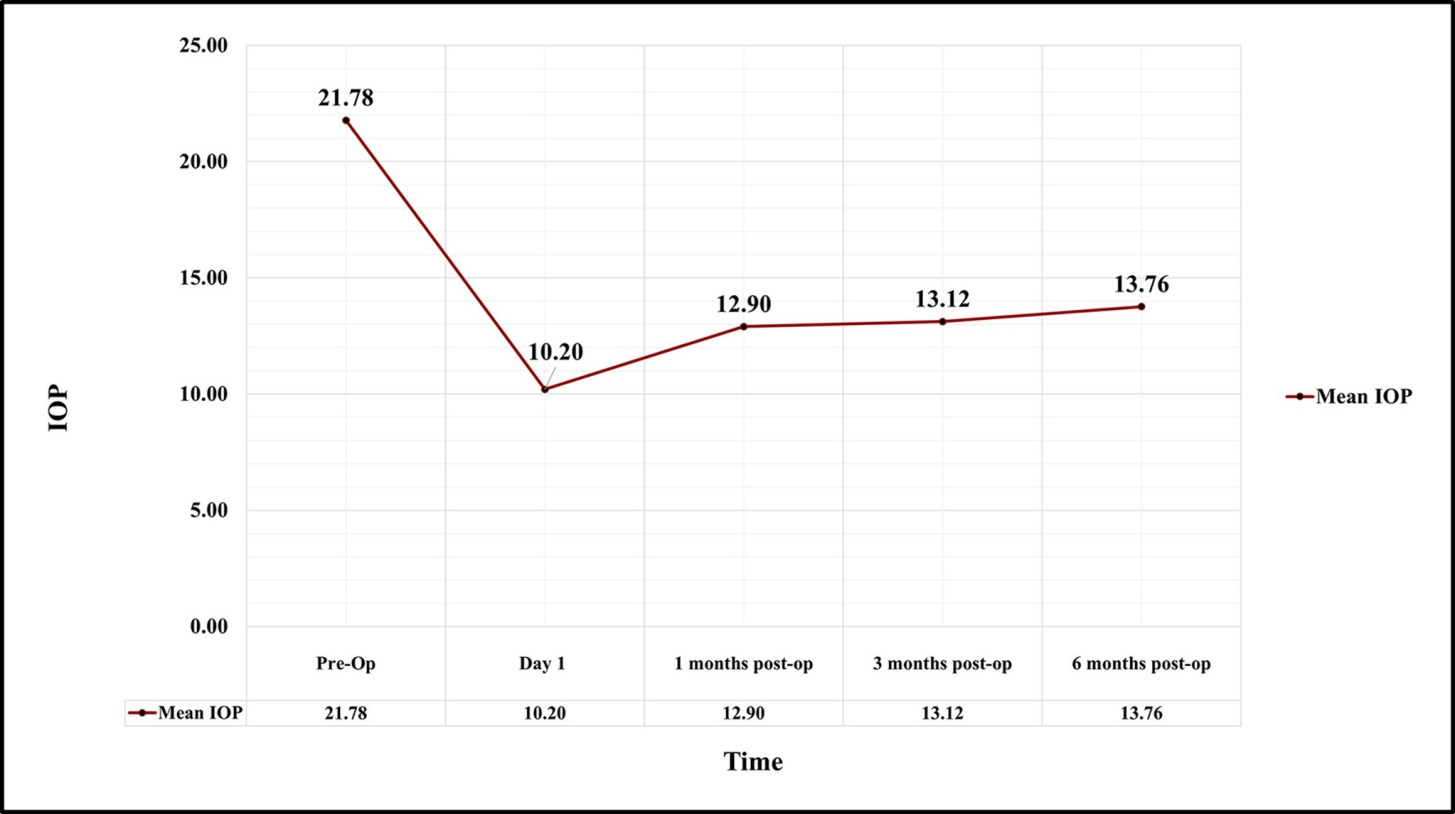
Displays preoperative and postoperative intraocular pressures. The mean IOP was 21.78 ± 7.62 preoperatively, 10.20 ± 5.26 at day 1 postoperatively, 12.9 ± 6.13 at 1 month postoperatively, 13.12 ± 3.9 at 3 months postoperatively, and 13.76 ± 3.95 at 6 months postoperatively.

Table 1 shows the surgical profile. Among the surgeries that the participants underwent, 35 patients (85.4%) underwent only deep sclerectomy, whereas six (14.6%) underwent deep sclerectomy and phacoemulsification. Regarding post-operative goniopuncture, only four patients (9.8%) required the procedure. Of these, one patient (25%) underwent goniopuncture at 1 month postoperatively, two patients (50%) at 3 months, and one patient (25%) at 4 months. Concerning the post-operative use of anti-glaucoma drops, only eight patients (19.5%) required medical therapy. Among them, five patients (62.5%) required a single medication, one patient (12.5%) required two medications, and two patients (25%) required three topical medications. Regarding the timing of the need for these drops postoperatively, three patients (37.5%) needed them 1 month postoperatively, one patient (12.5%) needed them 2 months postoperatively, two patients (25%) needed them 3 months postoperatively, and one patient (12.5%) needed them at 4 and 6 months postoperatively. Figure 1 displays the intraocular pressure pre-op and post-op. The mean IOP pre-op was 21.78 ± 7.62, day 1 post-op was 10.20 ± 5.26, 1 month post-op was 12.9 ± 6.13, 3 months post-op was 13.12 ± 3.9, and 6 months post-op was 13.76 ± 3.95.

**Table 1:** 

Surgery profile (*n* = 41)		
	*n*	%
Surgery		
Deep sclerectomy	35	85.4
Deep sclerectomy and phacoemulsification	6	14.6
Goniopuncture		
Yes	4	9.8
No	37	90.2
Timing after surgery (for Goniopuncture) (in months) (*n* = 4)		
1 month	1	25
3 months	2	50
4 months	1	25
6 months	0	0
Use of drops after surgery		
Yes	8	19.5
No	33	80.5
Numbers of drops		
1	5	62.5
2	1	12.5
3	2	25
Timing after surgery (for starting drops) (in months) (*n* = 8)		
1 month	3	37.5
2 months	1	12.5
3 months	2	25
4 months	1	12.5
6 months	1	12.5

Table 2 presents the outcome profile. Regarding the success rate of the intervention described previously, 1 patient (2.4%) experienced treatment failure, 7 patients (17.1%) had a qualified successful outcome, and 33 patients (80.5%) achieved complete success. In regard to preoperative visual acuity (VA) in the studied eye, 19 patients (51.35%) had a VA of 20/40 or better, 9 patients (24.32%) had a VA between 20/50 and 20/100, 4 patients (10.81%) had a VA of 20/200 or worse, and 5 patients (13.51%) (young preverbal patients) demonstrated fixation and following. At the final postoperative visit, 24 patients (66.67%) had a VA of 20/40 or better, 5 patients (13.89%) had a VA between 20/50 and 20/100, 4 patients (11.11%) had a VA of 20/200 or worse, and 3 patients (8.33%) (young preverbal patients) were fixating and following.

**Table 2:** 

Outcomes and visual acuity pre-op and post-op (final visit) (*n* = 41)		
	*n*	%
Success of intervention		
Failure	1	2.4
Qualified success	7	17.1
Complete success	33	80.5
Visual acuity pre-op (*n* = 37)		
20/40 or better	19	51.35
Between 20/50 and 20/100	9	24.32
20/200 or worse	4	10.81
Fix and follow	5	13.51
Visual acuity post-op (last visit) (*n* = 36)		
20/40 or better	24	66.67
Between 20/50 and 20/100	5	13.89
20/200 or worse	4	11.11
Fix and follow	3	8.33

Surgical technique of deep sclerectomy using STPT.

Surgical technique

**Figure 2.**
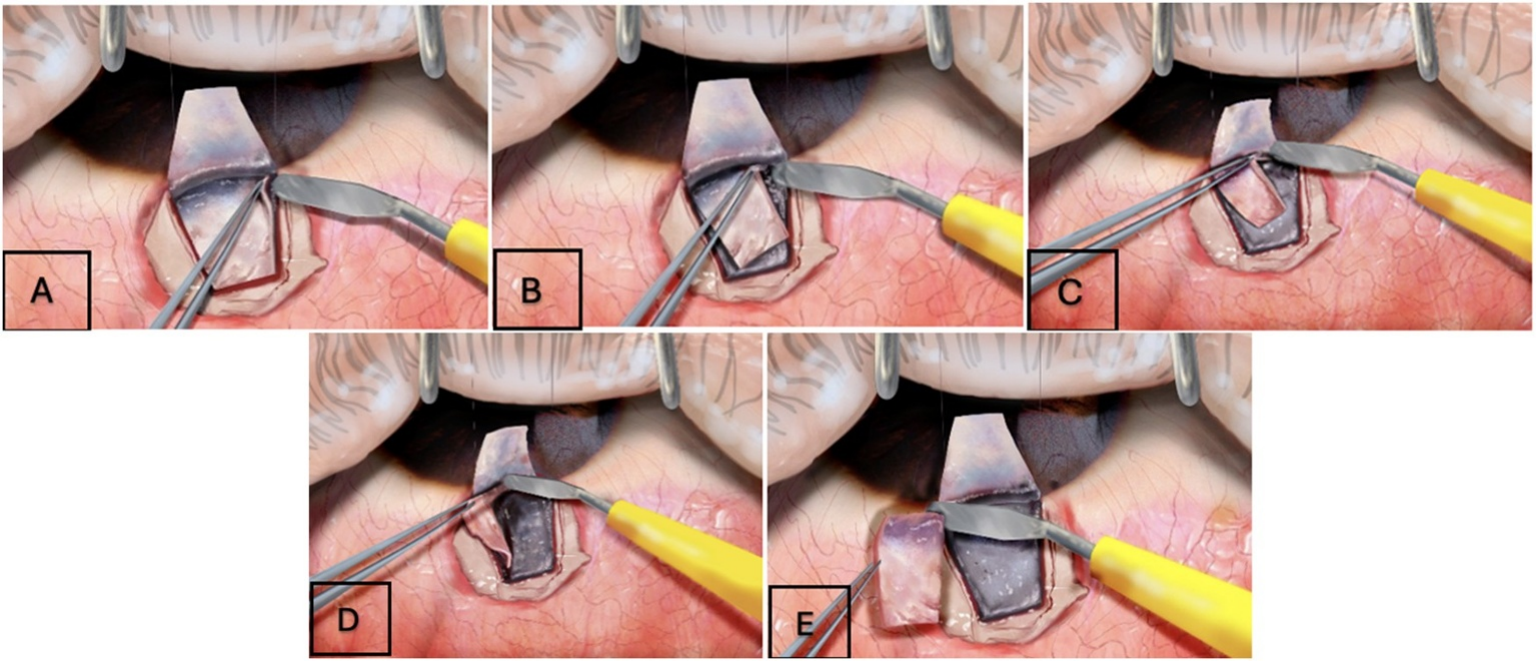
Illustration shows the STPT steps. The edge of the superficial scleral flap is dissected anteriorly, by 2 mm, into the clear corneal tissue using a 1.25 mm crescent knife (Mani®) (A). Using the side edge of a crescent knife, the flap is peeled transversely along a horizontal plane using controlled lateral traction (B). Peeling proceeds from side to side, sparing the TDW (C, D) until the final edge is reached and the flap is detached using the crescent knife (E), thereby eliminating the need for scissors.

**Figure 3.**
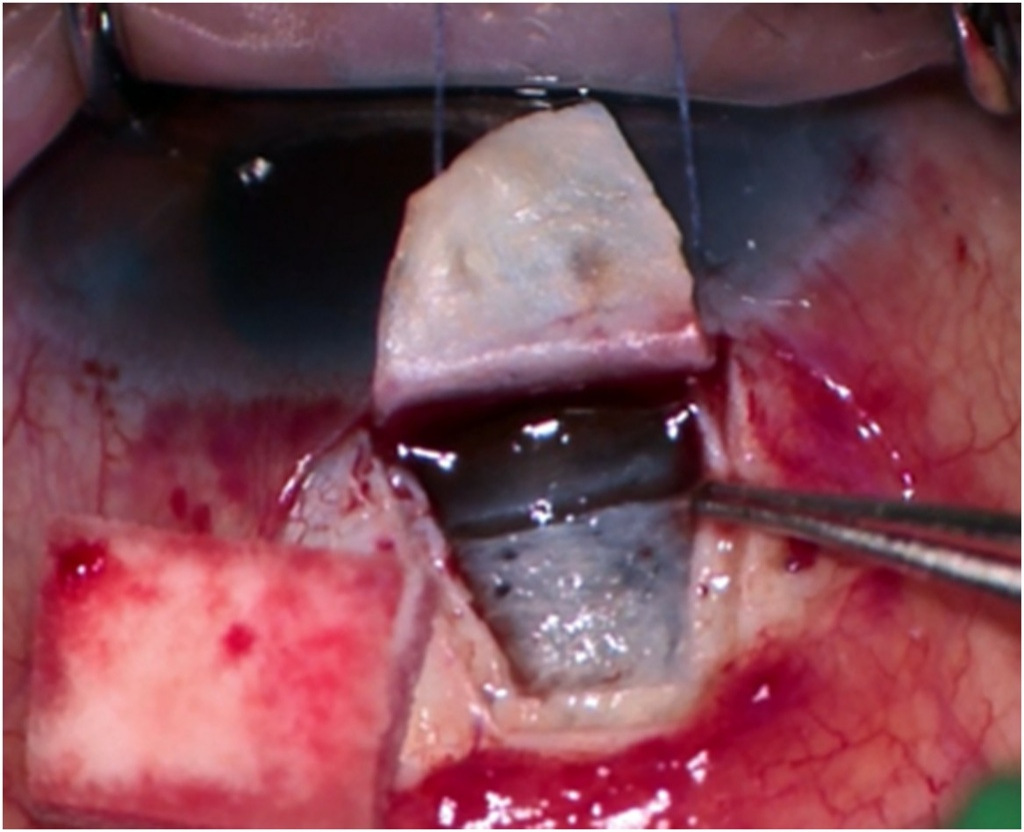
The resulting scleral bed in STPT is broader and smoother than when using traditional dissection.

1. Conjunctival and scleral exposure

- A fornix-based conjunctival flap was created.
- A superficial scleral flap (~5 × 5 mm, one-third scleral thickness) was dissected, extended 1–2 mm into the clear cornea, and then reflected anteriorly.
- Mitomycin C (MMC) at a concentration of 0.2 mg/mL was applied for 3.5 minutes beneath the created scleral flap within the conjunctival pocket and was followed by a thorough irrigation with the balanced salt solution (BSS).

2. Deep scleral flap dissection

- A deep scleral flap (~4 × 4 mm, about 90% of scleral thickness) was dissected beneath the superficial flap until Schlemm’s canal was unroofed.
- At this stage, the deep flap was not fully excised.

3. STPT

- The deep scleral flap was grasped with 0.12-mm toothed forceps.
- The edge of the superficial scleral flap was dissected anteriorly 2 mm into clear corneal tissue using a 1.25-mm crescent knife (Mani®) (Fig. 2A).
- Using the side edge of a crescent knife, the flap was peeled transversely and *not cut*, in a horizontal plane using controlled lateral traction. This maneuver preserves a thin, uniform layer over the TDM that is sufficiently strong to resist inadvertent penetration (Fig. 2B).
- This method replaces the conventional cutting approach that uses micro-scissors, which often leaves a long posterior stump that may cover Schlemm’s canal or risk damaging the TDM.
- Peeling proceeded from side to side (typically from right to left for right-handed surgeons), sparing the TDM (Fig. 2C and D) until the final edge was reached, and the flap was detached using the crescent knife (Fig. 2E), thus avoiding scissors entirely.
- Profuse percolation of the aqueous humor typically begins at this stage. If visualization is impaired, an air bubble is injected through paracentesis to temporarily reduce percolation.
- Any residual tissue within Schlemm’s canal was gently removed using fine forceps, such as Mermoud’s canal forceps.
- Compared to traditional dissection, the resulting scleral bed was broader and smoother (Fig. 3).
- The preserved TDM allowed for controlled and sustained aqueous percolation.

4. Optional viscocanalostomy

- A 28–32 G cannula was inserted into Schlemm’s canal to inject a viscoelastic agent (e.g., hyaluronic acid), enhancing circumferential canal dilation.
- Leaving a small amount of viscoelastic material in the scleral bed helps maintain space and reduces post-operative fibrosis.

5. Closure

- The superficial scleral flap was repositioned and loosely secured with 10–0 nylon sutures.
- The conjunctiva was closed in a watertight manner.

A supplementary video demonstrating the steps of STPT is available in the supplementary material (Supplemental Digital Content Video, available at: http://links.lww.com/MS9/B280).

## Discussion

Traditional glaucoma management typically reserves surgery as a last resort, to be adopted only after all other attempted medical and laser treatments. This approach stems from the relatively high complication rates and unpredictability associated with trabeculectomy, including bleb leaks, bleb-associated infections, and hypotony^[^5^]^. Deep sclerectomy is associated with specific potential complications, including intraoperative rupture of the TDM, postoperative ocular hypertension that may necessitate laser goniopuncture, and fibrosis or failure of the filtering bleb. However, owing to its lower complication rates, it can be considered at an earlier stage of the disease, even as a first-line treatment when medical or laser options are inadequate or unavailable^[^8–10^]^. DS targets the site with the highest outflow resistance in open-angle glaucoma, specifically the inner wall of Schlemm’s canal and the juxtacanalicular trabecular meshwork^[^7,11^]^. DS is indicated for both primary and secondary open-angle glaucoma. Additionally, DS is an appropriate surgical option for uveitic glaucoma, as it induces less postoperative inflammation compared with penetrating procedures^[^12^]^. Patients with high myopia, who face an increased risk of choroidal detachment, also benefit from the better-controlled IOP reduction achieved using DS^[^13^]^. The effectiveness of non-invasive procedures has traditionally been considered inferior to that of trabeculectomy. However, various adjunctive techniques have been introduced to increase the success rate, including the intraoperative application of antimetabolites, the use of implants, and early laser goniopuncture when the IOP exceeds the target level^[^5,14^]^. DS reportedly achieves good IOP control in the early postoperative period^[^5^]^. Previous studies have shown that the complete success rate for DS ranges between 53.1 and 71.8% at 6 months^[^15,16^]^. In this study, the complete success rate was 80.5% at 6 months when the new modified dissection technique, STPT, was used for DS, which indicates a stronger IOP-lowering effect. Deep sclerectomy can also be combined with phacoemulsification without compromising patient outcomes. The advantage of STPT over traditional DS is the ability of the former to widen the surface area of the TDM, based on the assumption that a larger TDM is better, which in turn enhances the outflow of aqueous fluid.

Laser goniopuncture (LGP) is an Nd:YAG laser that improves long-term surgical success owing to its perforation of the TDM and the subsequent increase in aqueous outflow. However, goniopuncture converts non-invasive surgery into invasive surgery, and penetration without peripheral iridectomy may lead to iris incarceration in the ostia of the TDM^[^17^]^. LGP is considered an important adjunct to DS when low-target IOPs have to be achieved, and the reported rates vary from 20% to 72% for the LGP needed after deep sclerectomy surgery^[^18–20^]^. In this study, the percentage of patients who needed LGP after STPT was low (12.2%).

DS surgery with STPT modification was useful for achieving better-controlled IOP and reducing the need for further interventions, with no reported complications. However, this study has several limitations. The results indicate short-term efficacy of the intervention, and there is a need for a longer follow-up period and a larger sample size in future investigations evaluating post-operative IOP control and surgical outcomes. Additionally, a methodological limitation of this study is the inclusion of both pediatric and adult glaucoma patients within a single analytical cohort, which may introduce heterogeneity and affect the interpretability and generalizability of the results. Future studies with larger sample sizes, extended follow-up, and appropriate population stratification are warranted.

## Conclusion

This study highlights the efficacy of STPT, a novel modification of traditional non-invasive deep sclerectomy. STPT is based on the controlled transverse peeling of the deep scleral flap to create a broader and smoother scleral bed, thereby achieving enhanced aqueous humor percolation. The clinical application of this technique has led to better IOP control and a reduced need for post-operative antiglaucomatous medications and goniopuncture. These outcomes suggest that STPT could be a safer and more effective approach to enhance the effectiveness of deep sclerectomy, especially in cases where sustained filtration with minimal risk of anterior chamber compromise is desired. Nevertheless, the study is limited by its short-term outcomes. Future studies incorporating larger cohorts and extended follow-up periods are warranted to further evaluate long-term postoperative intraocular pressure control and overall surgical outcomes.

## Data Availability

Data are available from the corresponding author upon reasonable request.

## Research registration unique identifying number (UIN)

ResearchRegistry11441.

## Précis

Sirhy’s transverse peeling technique significantly lowers intraocular pressure in glaucoma patients, achieving a complete success rate of 80.5%and reducing the need for medications and additional procedures, making it a promising deep sclerectomy modification.
